# Performance of the Newly Proposed Peguero-Lo Presti Criterion in Adults with Hypertrophic Cardiomyopathy

**DOI:** 10.31083/j.rcm2309319

**Published:** 2022-09-16

**Authors:** Yiwei Cao, Lei Liang, Xiaowei Yao, Xiling Shou, Gong Cheng, Jianli Fu, Haoyu Wu

**Affiliations:** ^1^Department of Electrocardiology, Shaanxi Provincial People's Hospital, 710068 Xi'an, Shaanxi, China; ^2^Department of Cardiology, Shaanxi Provincial People's Hospital, 710068 Xi'an, Shaanxi, China; ^3^Department of Ultrasonic Diagnosis Center, Shaanxi Provincial People’s Hospital, 710068 Xi'an, Shaanxi, China

**Keywords:** Peguero-Lo Presti criterion, electrocardiogram criteria, hypertrophic cardiomyopathy, sensitivity

## Abstract

**Background:**

The classic electrocardiogram (ECG) criteria have been applied to left ventricular hypertrophy (LVH) screening but have low sensitivity. Recently, the newly proposed Peguero-Lo Presti criterion has been proven to be more sensitive in detecting LVH in patients with hypertension than several current ECG criteria. The diagnostic value of the Peguero-Lo Presti criterion in hypertrophic cardiomyopathy (HCM) patients has not been fully evaluated. This study aims to test whether the new Peguero-Lo Presti criterion can improve the diagnostic performance in patients with HCM.

**Methods:**

This study included HCM patients and sex-and age-matched healthy control subjects. The diagnostic performance of the Peguero-Lo Presti criterion was evaluated along with the Sokolow-Lyon criterion, Cornell criterion, and total 12-lead voltage criterion.

**Results:**

Overall, 63 HCM patients and 63 controls were enrolled. The diagnostic accuracy, sensitivity and specificity of Peguero-Lo Presti criterion were 74.6%, 73.0% and 76.2%, respectively. The Peguero-Lo Presti criterion had the highest sensitivity, while the Cornell criterion and Sokolow-Lyon criterion had the highest specificity (96.8%). The area under the curve (AUC) showed that the Peguero-Lo Presti criterion was 0.809 (95% CI, 0.730–0.874; *p <* 0.0001), Sokolow-Lyon criterion was 0.841 (95% CI, 0.766–0.900) and total 12-lead voltage criterion was 0.814 (95% CI, 0.735–0.878). There was no significant difference in AUC between Peguero-Lo Presti criterion and Sokolow-Lyon criterion (*p =* 0.533), or Peguero-Lo Presti criterion and total 12-lead voltage criterion (*p =* 0.908). Receiver operator characteristic curve analysis of the Peguero-Lo Presti criterion showed an optimal cutoff of >3.15 mV for men (sensitivity: 63.9%; specificity: 80.0%) and >2.29 mV for women (sensitivity: 78.6%; specificity: 85.7%).

**Conclusions:**

The Peguero-Lo Presti criterion provides high sensitivity for ECG diagnosis of HCM patients and can be considered when applicable but this needs to be verified in a larger population.

## 1. Introduction

Left ventricular hypertrophy (LVH) is considered to be a major predictor of cardiovascular events [1, 2]. It has been reported that regardless of whether a patient is suffering from hypertension, LVH diagnosed by electrocardiogram (ECG) is strongly associated with cardiovascular morbidity and mortality [3, 4, 5]. Therefore, it is necessary to diagnose LVH as soon as possible for further examination and treatment. Echocardiography is considered as a central cardiac imaging modality for LVH diagnosis and monitoring [6]. However, ECG is also an important screening method for LVH detection because of its ease of use, wide availability and proven independent clinical prognostic impact. Some ECG criteria for LVH detection have been proposed. However, these ECG criteria have many limitations in clinical use because the electrocardiographic indicators of LVH are relatively insensitive. The sensitivity of these criteria also varies with the various etiologies of LVH [7]. Although many classical ECG voltage criteria are also used to screen hypertrophic cardiomyopathy (HCM), the overall reliability of these criteria is low [8]. Therefore, new criteria need to be developed to reduce the rate of false-negative screening.

Peguero *et al*. [9] proposed a novel ECG voltage criterion (Peguero-Lo Presti criterion) for identifying LVH with better sensitivity than several classical ECG voltage criteria in a group of patients with hypertension. Since then, the new criterion has been tested in several studies [10, 11, 12] for LVH detection in patients with hypertension but it has not been fully evaluated in the population of HCM. Therefore, the aim of this study is to test whether the Peguero-Lo Presti criterion can improve the diagnostic accuracy of HCM.

## 2. Materials and Methods

### 2.1 Study Design and Study Population

This study included HCM patients who were hospitalized in our hospital from February 2019 to June 2021. In the same period, we selected sex-and age-matched healthy subjects as the controls. These healthy subjects were selected from a database who underwent regular physical examinations. Transthoracic echocardiography was used to diagnose HCM according to the ESC guidelines [6]. HCM was defined by a wall thickness ≥15 mm in one or more left ventricular myocardial segments measured by echocardiography that was not explained solely by loading conditions [6]. Echocardiography was performed by two experienced echocardiographers who were blinded to clinical data and ECG results using GE Vivid E95 ultrasound device (GE Medical Systems, Milwaukee, WI, USA) in accordance with the guideline [13]. Echocardiographers with more than 20 years of working experience had received standardized training and obtained certificates in echocardiography. The exclusion criteria included age <18 years, previous myocardial infarction, ECG or echocardiography indicating myocardial infarction, ventricular paced rhythm, atrioventricular block, bundle branch block, ventricular arrhythmias, Wolff-Parkinson-White syndrome, hypertension, or another type of structural heart disease that could cause LVH. Subjects with incomplete data, poor quality echocardiogram or ECG were also excluded.

### 2.2 Data Collection

Data collection was performed using standardized questionnaires. Height and weight were measured by trained technicians while the patient was barefoot and wearing light clothing. Dyslipidemia was defined as triglyceride ≥150 mg/dL, total cholesterol ≥220 mg/dL, low-density lipoprotein cholesterol ≥140 mg/dL, or receiving medication. Atrial fibrillation was defined as the patient’s current ECG results. Coronary artery disease was defined as one or more major coronary arteries with diameter stenosis of 50% or more confirmed by coronary angiography or coronary computed tomographic angiography. Stroke was defined as focal or systemic neurological dysfunction lasting more than 24 hours caused by acute cerebrovascular events, which was confirmed by clinical and radiological examination. Laboratory tests, including fasting blood glucose, hemoglobin, serum urea, creatinine, and uric acid, were measured after overnight fasting by an automatic biochemical analyser (7180; Hitachi, Tokyo, Japan).

### 2.3 ECG Analysis

A standard 12-lead ECG (1 mV/10 mm and 25 mm/s) was performed for each subject at rest by trained technicians on the same day as the echocardiography. ECG interpretations were independently assessed by two experienced cardiologists who had more than 15 years of work experience and did not know the echocardiographic data. ECG measurements were performed manually with calipers. Inconsistent ECG interpretation results were reconciled through consensus. The newly proposed Peguero-Lo Presti criterion was obtained by adding SD (the amplitude of the deepest S wave in any lead) to the S amplitude in V4 (SD + SV4). The cutoff values were ≥2.3 mV for women and ≥2.8 mV for men [9]. We also assessed the following 3 classic ECG screening algorithms: Cornell criterion (RaVL + SV3, for men >2.8 mV, for women >2.0 mV) [14], Sokolow-Lyon criterion (SV1 + RV5/RV6 ≥3.5 mV) [14], and total 12-lead voltage criterion (R wave to the nadir of the Q/S wave >17.5 mV) [14].

### 2.4 Statistical Analysis

The normality of the distribution of continuous variables was tested by the Kolmogorov-Smirnov test. Continuous variables were presented as median (interquartile range) or mean ± standard deviation, depending on non-normal or normal distribution of the data. Categorical variables were presented as frequencies and percentages. The chi-square test, Student’s *t*-test, or Mann-Whitney test was used to compare differences as appropriate. Accuracy, sensitivity, specificity, positive predictive value (PPV), and negative predictive value (NPV) were used to detect the diagnostic performance of the ECG criteria. The agreement between the ECG criteria and the reference standard was evaluated by the McNemar test [15]. Receiver operating characteristic (ROC) curves were used to assess the predicted performance for the ECG criteria and to assess the best cutoff values for the Peguero-Lo Presti criterion. The area under the ROC curve (AUC) was used to determine the ECG criteria as a metric of overall diagnostic performance, and statistical comparisons of AUCs in the analyses of ROC curves were performed using Hanley and McNeil formula [16]. All analyses were performed with MedCalc 20.0 (MedCalc Software, Ostend, Belgium). A *p* value < 0.05 was considered to be statistically significant.

Using a two-sided z-test at a significance level of 0.05, it was estimated that a sample of 17 patients with HCM and 17 controls achieved 92% power to detect a difference of 0.3 between AUC under the null hypothesis of 0.5 and AUC under the alternative hypothesis of 0.8.

## 3. Results

### 3.1 Clinical Characteristics of This Study

A total of 63 HCM (35 men; mean age 60.1 ± 13.9 years) and 63 sex- and age-matched controls (35 men; mean age 59.1 ± 13.0 years) were enrolled. There were no significant differences in age, body mass index, body surface area, systolic blood pressure, or diastolic blood pressure (*p >* 0.05). Laboratory tests revealed that the HCM group had higher serum urea and creatinine (*p <* 0.05), while fasting blood glucose, hemoglobin, and uric acid did not differ significantly (*p >* 0.05) (Table 1).

**Table 1.** **Clinical characteristics of patients in this study**.

Parameter		HCM (N = 63)	Controls (N = 63)	*p*
Age, yrs		60.1 ± 13.9	59.1 ± 13.0	0.653
Body mass index, ${\mathrm{kg/m}}^{2}$		24.6 ± 3.0	24.8 ± 3.2	0.695
Body surface area, $m^{2}$		1.7 ± 0.2	1.7 ± 0.2	0.758
Systolic blood pressure, mmHg		124.4 ± 11.2	127.9 ± 12.2	0.101
Diastolic blood pressure, mmHg		78.7 ± 10.7	82.1 ± 11.5	0.087
Diabetes mellitus, n (%)		13 (20.6)	-	-
Dyslipidemia, n (%)		10 (15.9)	-	-
Atrial fibrillation, n (%)		9 (14.3)	-	-
Coronary artery disease, n (%)		19 (30.2)	-	-
Stroke, n (%)		10 (15.9)	-	-
Laboratory tests				
	Fasting blood glucose, mmol/L	5.4 ± 1.3	5.5 ± 1.2	0.620
	Hemoglobin, %	6.0 ± 0.7	6.1 ± 0.9	0.521
	Serum urea, mmol/L	6.4 ± 2.1	5.4 ± 1.3	0.002
	Creatinine, µmol/L	69.4 ± 22.7	62.4 ± 12.8	0.033
	Uric acid, µmol/L	348.6 ± 122.0	333.1 ± 91.8	0.424
ECG criteria				
	Cornell criterion (RaVL + SV3), mV	2.4 ± 1.3	1.4 ± 0.6	<0.001
	Sokolow-Lyon criterion (SV1 + RV5/RV6), mV	4.1 ± 1.8	2.3 ± 0.6	<0.001
	Total 12-lead voltage criterion (R wave to the nadir of the Q/S wave), mV	22.5 ± 7.4	14.9 ± 3.0	<0.001
	Peguero-Lo Presti criterion (SD + SV4), mV	3.6 ± 1.5	2.1 ± 0.9	<0.001

Data are presented as the mean ± standard deviation or percentages (%). HCM, hypertrophic cardiomyopathy; ECG, electrocardiogram.

Electrocardiographic analysis revealed that the HCM group had higher values than the control group using the Cornell criterion (2.4 ± 1.3 mV vs. 1.4 ± 0.6 mV, *p <* 0.001), Sokolow-Lyon criterion (4.1 ± 1.8 mV vs. 2.3 ± 0.6 mV, *p <* 0.001), total 12-lead voltage criterion (22.5 ± 7.4 mV vs. 14.9 ± 3.0 mV, *p <* 0.001) and Peguero-Lo Presti criterion (3.6 ± 1.5 mV vs. 2.1 ± 0.9 mV, *p <* 0.001) (Table 1).

### 3.2 Diagnostic Performance of ECG Criteria in Patients with HCM

The diagnostic accuracy, sensitivity and specificity of the Peguero-Lo Presti criterion were 74.6%, 73.0% and 76.2%, respectively. Furthermore, the Peguero-Lo Presti criterion did not show a lack of agreement with the reference standard. Among the four ECG criteria, the Peguero-Lo Presti criterion had the highest sensitivity, while the Cornell criterion and Sokolow-Lyon criterion had the highest specificity (96.8%). The Sokolow-Lyon criterion and total 12-lead voltage criterion had the highest accuracy (78.6%) (Table 2).

**Table 2.** **Diagnostic performance of the four ECG criteria in patients with HCM**.

ECG criteria	Accuracy (%)	Sensitivity (%)	Specificity (%)	PPV (%)	NPV (%)	McNemar Test*
Cornell criterion	71.4 (62.7–79.1)	46.0 (33.4–59.1)	96.8 (89.0–99.6)	93.6 (78.3–98.3)	64.2 (58.7–69.3)	<0.0001
Sokolow-Lyon criterion	78.6 (70.4–85.4)	60.3(47.2–72.4)	96.8 (89.0–99.6)	95.0 (82.7–98.7)	70.9 (64.2–76.9)	<0.0001
Total 12-lead voltage criterion	78.6 (70.4–85.4)	71.4(58.6–82.1)	85.7 (74.6–93.3)	83.3 (72.8–90.3)	75.0 (66.7–81.8)	0.122
Peguero-Lo Presti criterion	74.6 (66.1–81.9)	73.0(60.4–83.4)	76.2 (63.8–86.0)	75.4 (65.8–83.0)	73.9 (64.8–81.3)	0.860

*A *p* value < 0.05 indicates lack of agreement. The null hypothesis is that the ECG criterion has agreement with the reference standard. ECG, electrocardiogram; PPV, positive predictive value; NPV, negative predictive value.

**Fig. 1.**
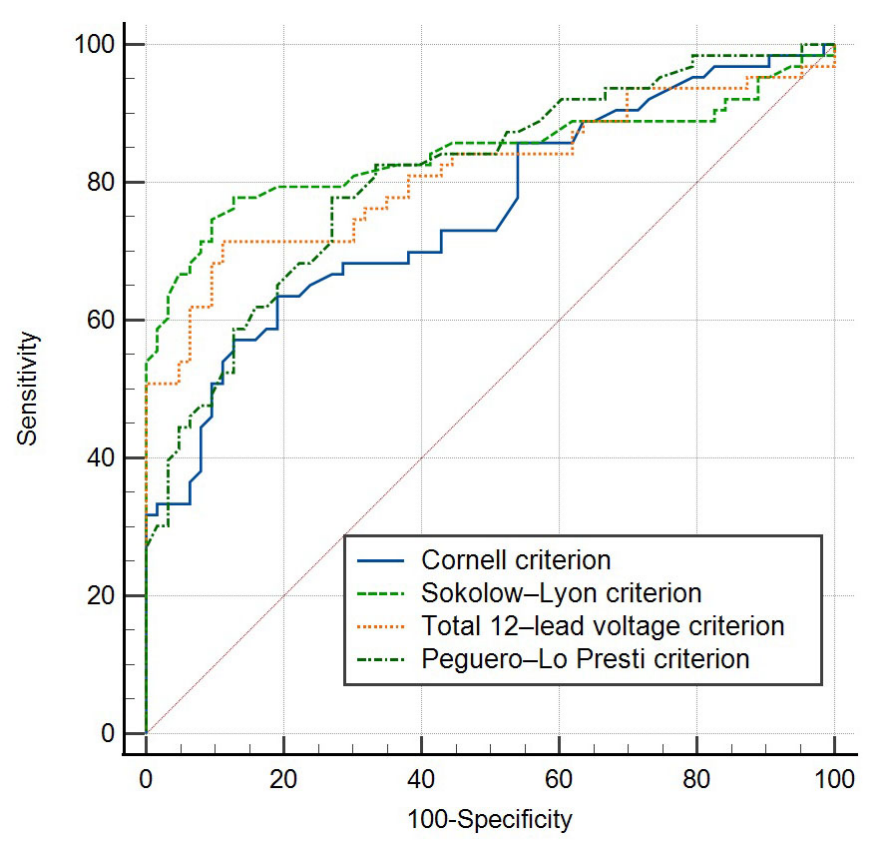
**ROC curve of the four ECG criteria in patients with HCM**.

The ROC curve was also performed. The AUC for the Peguero-Lo Presti criterion was 0.809 (95% CI, 0.730–0.874; *p <* 0.0001). Among the four ECG criteria, the highest AUC was found with the Sokolow-Lyon criterion (AUC: 0.841; 95% CI, 0.766–0.900; *p *< 0.0001) (Table 3; Fig. 1). There was no significant difference in AUC between Peguero-Lo Presti criterion and Sokolow-Lyon criterion (*p =* 0.533), or Peguero-Lo Presti criterion and total 12-lead voltage criterion (*p =* 0.908).

**Table 3.** **AUC values of the four ECG criteria in patients with HCM**.

ECG criteria	AUC	95% CI	p*
Cornell criterion	0.758	0.673–0.830	<0.0001
Sokolow-Lyon criterion	0.841	0.766–0.900	<0.0001
Total 12-lead voltage criterion	0.814	0.735–0.878	<0.0001
Peguero-Lo Presti criterion	0.809	0.730–0.874	<0.0001

*The null hypothesis is that the AUC is 0.5. ECG, electrocardiogram; AUC, area under the ROC curve; CI, confidence interval.

### 3.3 Diagnostic Performance of the Peguero-Lo Presti Criterion in Men and Women with HCM

**Fig. 2.**
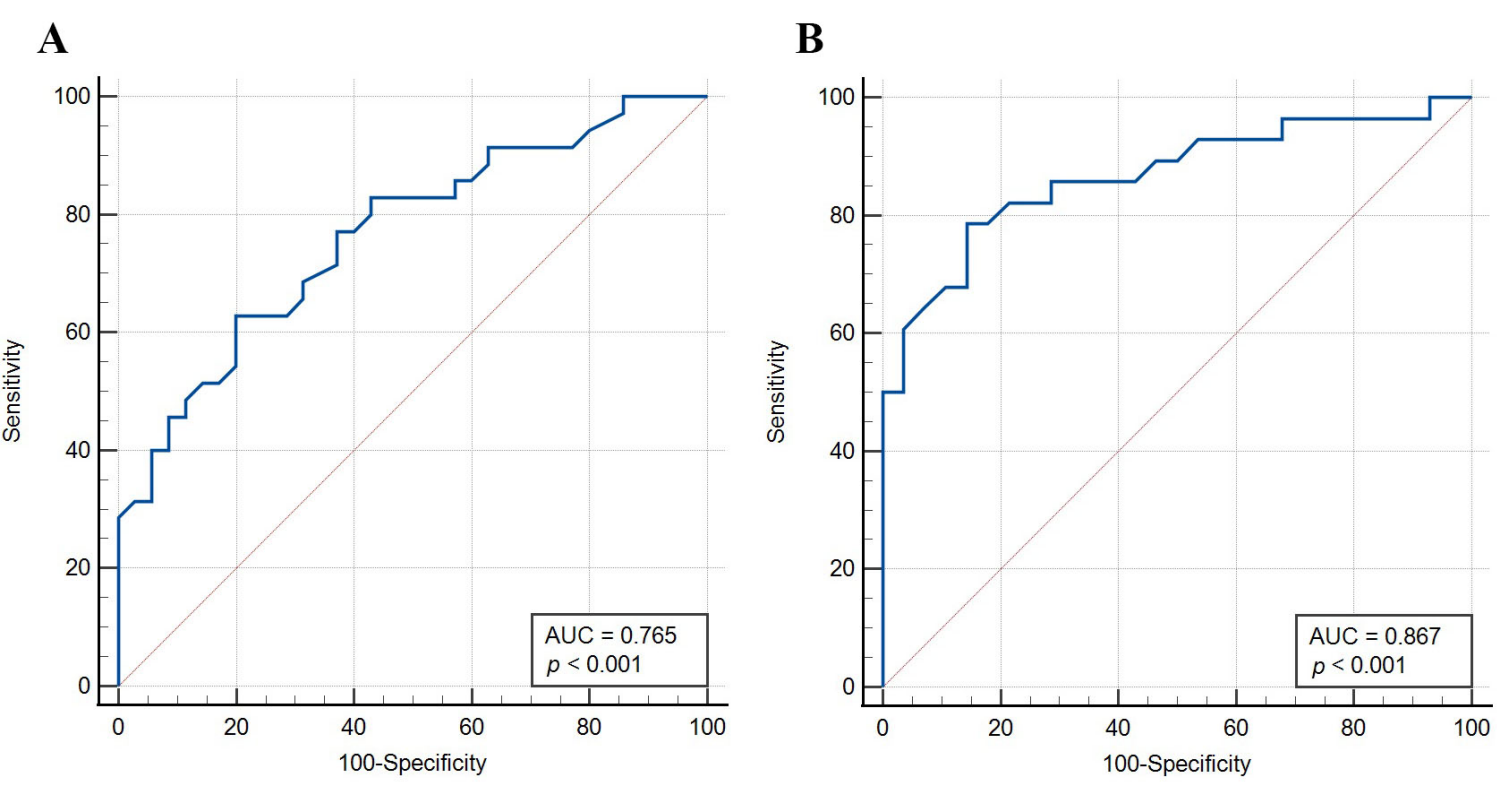
**ROC curve of the Peguero-Lo Presti criterion in men (A) and women (B) with HCM**.

Compared with men, the Peguero-Lo Presti criterion for women had higher diagnostic accuracy (71.4% for men; 82.1% for women), sensitivity (68.6% for men; 78.6% for women), specificity (74.3% for men; 85.7% for women), PPV (72.7% for men; 84.6% for women), and NPV (70.3% for men; 80.0% for women) (Table 4). The AUCs for men and women were 0.765 (*p *< 0.001) and 0.867 (*p *< 0.001), respectively (Fig. 2). The optimal cutoff value of the ROC curve was determined according to the maximum Youden index. The optimal cutoff value for men was >3.15 mV (sensitivity: 63.7%; specificity: 80.0%). The optimal cutoff value for women was >2.29 mV (sensitivity: 78.6%; specificity: 85.7%).

**Table 4.** **Diagnostic performance of the Peguero-Lo Presti criterion in men and women with HCM**.

Sex	Accuracy (%)	Sensitivity (%)	Specificity (%)	PPV (%)	NPV (%)	McNemar Test*
Men	71.4 (59.4–81.6)	68.6 (50.7–83.2)	74.3 (56.7–87.5)	72.7 (59.3–83.0)	70.3 (58.3–80.0)	0.824
Women	82.1 (69.6–91.1)	78.6 (59.1–91.7)	85.7 (67.3–96.0)	84.6 (68.5–93.3)	80.0 (66.0–89.2)	0.754

*A *p* value < 0.05 indicates a lack of agreement. The null hypothesis is that the ECG criterion has agreement with the reference standard. PPV, positive predictive value; NPV, negative predictive value.

## 4. Discussion

LVH is an important manifestation of preclinical cardiovascular disease, which can significantly predict cardiovascular events [17]. It has been reported that ECG-based diagnostic criteria are better than cardiovascular magnetic resonance imaging in predicting cardiovascular events [18]. Many ECG criteria for LVH have been proposed and used clinically, and those most commonly used are Sokolow-Lyon criterion and Cornell criterion [19]. However, these criteria have the characteristics of high specificity and low sensitivity. For example, the Sokolow-Lyon criterion has a median sensitivity of 21% (4%–52%) and specificity of 89% (53%–100%) [20]. The specificity of the Cornell criterion is approximately 90%, while the sensitivity is only 20%–40% [9, 21]. The performance of ECG for LVH detection is affected by several factors. In general, ECG evaluates the presence of LVH by detecting electrical voltage changes caused by an increased left ventricular mass. However, the electrical voltage is also affected by the myocardial interstitium (such as fibrosis and other material deposition), cardiac conduction abnormalities, left ventricular geometry, pulmonary diseases, and the distance between the heart and the electrodes [22]. Other factors affecting the results include sex and race [23]. Therefore, it is particularly urgent to propose a new ECG criterion with higher sensitivity for use in the clinic.

Peguero *et al*. [9] recently proposed a novel ECG voltage criterion (Peguero-Lo Presti criterion) for LVH detection. The Peguero-Lo Presti criterion was obtained by adding SD (the amplitude of the deepest S wave in any lead) to the S amplitude in V4 (SD + SV4) [9]. They found that the new criterion improved the sensitivity of LVH detection in patients with hypertension while maintaining sufficient specificity. The results also showed that the Peguero-Lo Presti criterion had higher diagnostic accuracy than the Sokolow-Lyon criterion and Cornell criterion. Since then, the new criterion has been validated in several studies [10, 11, 12] for LVH detection in patients with hypertension.

The Peguero-Lo Presti criterion has not been fully evaluated in patients with HCM. Tiron *et al*. [24] found that compared with Sokolow-Lyon criterion and Cornell criterion, Peguero-Lo Presti criterion was the only criterion related to both left ventricular mass index and maximum thickness in HCM patients. In this study, the Peguero-Lo Presti criterion was used to screen HCM and was compared with other commonly used ECG criteria. Sensitivity and specificity are classical parameters to characterize a diagnostic test. Sensitivity refers to the percentage of patients correctly classified in the diseased category and the specificity refers to the percentage of patients correctly classified in the non-diseased category. For screening tests, sensitivity would be favored over specificity, while for confirmatory tests, specificity would be favored over sensitivity [25]. As a screening test, the results of this study showed that the Peguero-Lo Presti criterion had the highest sensitivity (73.0%), followed by the total 12-lead voltage criterion (71.4%). The Cornell criterion and Sokolow-Lyon criterion were relatively insensitive (46.0% and 60.3%, respectively). However, compared with other ECG criteria, the specificity of the Peguero-Lo Presti criterion was relatively low (76.2%). ROC curve is a graph of sensitivity (Y-axis) versus false-positive rate (1 – specificity) (X-axis), which can be used to summarize the overall accuracy of the diagnostic test. AUC calculated according to the ROC curve is a common index to measure the accuracy of a diagnostic test. The value of AUC can be between 0.5 and 1.0. Ideally, AUC of 1.0 represents a completely accurate test, while the AUC along the diagonal line in the graph is 0.5 that is no better than flipping a coin [25]. The ROC curve demonstrated that the Peguero-Lo Presti criterion had an AUC of 0.809, indicating its good overall performance. Therefore, the overall diagnostic accuracy of the Peguero-Lo Presti criterion is reliable for HCM. Recently, Gamrat *et al*. [26] applied the Peguero-Lo Presti criterion to detect LVH in patients with severe aortic stenosis. The results showed that the Peguero-Lo Presti criterion had improved sensitivity (55% vs. 9%–34%) and decreased specificity (72% vs. 78%–100%) for the detection of LVH compared with 8 single traditional ECG criteria. Compared with the traditional ECG-LVH criteria, the agreement between Peguero-Lo Presti criterion and echocardiographic LVH in patients with severe aortic stenosis was slightly better [26]. Matusik *et al*. [27] concluded that the Peguero-Lo Presti criterion and Cornell criterion were sex-specific and could provide the highest level of diagnostic accuracy. When screening for LVH in patients with cardiovascular diseases, routine use of Peguero-Lo Presti criterion should be considered [27]. Besides, Matusik *et al*. [28] tested a novel screening tool (${\mathrm{CAR}}_{2}$$E_{2}$ score) for LVH screening based on a point system including heart failure (1 point), age ≥40 years (1 point), chest radiograph indicating cardiac enlargement (2 points) and positive Peguero-Lo Presti criterion (2 points). The results showed that ${\mathrm{CAR}}_{2}$$E_{2}$ score ≥3 points had the best sensitivity for screening for LVH. ${\mathrm{CAR}}_{2}$$E_{2}$ score may improve prediction of LVH compared to other approaches [28].

We also compared the diagnostic performance of the Peguero-Lo Presti criterion between men and women. Compared with men, the Peguero-Lo Presti criterion for women had higher diagnostic accuracy (71.4% for men; 82.1% for women), sensitivity (68.6% for men; 78.6% for women), and specificity (74.3% for men; 85.7% for women). To improve the diagnostic accuracy of the Peguero-Lo Presti criterion in patients with HCM, we also calculated the optimal cutoff value of the ROC curve based on the proposed sensitivity and specificity to be determined by the maximum Youden index. The optimal cutoff value for men was >3.15 mV, and for women, it was >2.29 mV. We found that the optimal cutoff value for women is very close to the Peguero-Lo Presti criterion. Therefore, the Peguero-Lo Presti criterion may be more suitable for female patients with HCM.

The Peguero-Lo Presti criterion has been proven to be sensitive (62%) while maintaining high specificity (90%) in the detection of LVH in patients with hypertension [9]. However, several studies have reported different results. The applicability of the Peguero-Lo Presti criterion is heterogeneous, especially in Asian populations, with relatively reduced specificity and AUC for LVH detection in patients with hypertension, which may be attributed to the different ECG characteristics of different races and the characteristics of specific study populations [29, 30]. Therefore, although the results of this study suggest that the novel criterion may be a suitable ECG screening tool for patients with HCM, a larger population and further adjustments may be needed, including consideration of extra cardiac factors such as race and sex [31].

We should recognize that there are some limitations of this study. First, the sample size of this study was relatively small, and this was a single-center study. Many large-scale studies are required to verify the accuracy of the Peguero-Lo Presti criterion in patients with HCM. Second, we only compared the Peguero-Lo Presti criterion with the Cornell criterion, Sokolow-Lyon criterion and total 12-lead voltage criterion. Further studies including more ECG diagnostic criteria are needed. Third, the diagnosis of HCM was evaluated by two-dimensional echocardiography in this study, whereas cardiovascular magnetic resonance imaging can provide more detailed information about the structure and function of the heart. Nonetheless, echocardiography is considered a central cardiac imaging modality for the diagnosis and monitoring of HCM with good reproducibility and is still the most commonly used method. We should also recognize that cardiovascular magnetic resonance imaging is difficult to apply widely due to its lack of availability and cost. Finally, our study excluded some patients with specific conditions, such as bundle branch block, so the diagnostic value of the Peguero-Lo Presti criterion for this population is unknown.

## 5. Conclusions

The newly proposed Peguero-Lo Presti criterion provides high sensitivity for ECG diagnosis in HCM patients and can be considered when applicable but it needs to be verified in a larger population.
